# Nanoparticles in the clinic: An update

**DOI:** 10.1002/btm2.10143

**Published:** 2019-09-05

**Authors:** Aaron C. Anselmo, Samir Mitragotri

**Affiliations:** ^1^ Division of Pharmacoengineering and Molecular Pharmaceutics, Eshelman School of Pharmacy University of North Carolina at Chapel Hill Chapel Hill North Carolina; ^2^ John A Paulson School of Engineering and Applied Sciences Harvard University, Wyss Institute of Biologically Inspired Engineering Cambridge Massachusetts

**Keywords:** clinic, clinical translation, clinical trials, drug delivery, nanomedicine, nanoparticles, translational medicine

## Abstract

Nanoparticle drug delivery systems have been used in the clinic since the early 1990's. Since that time, the field of nanomedicine has evolved alongside growing technological needs to improve the delivery of various therapeutics. Over these past decades, newer generations of nanoparticles have emerged that are capable of performing additional delivery functions that can enable treatment via new therapeutic modalities. In the current clinical landscape, many of these new generation nanoparticles have reached clinical trials and have been approved for various indications. In the first issue of *Bioengineering & Translational Medicine* in 2016, we reviewed the history, current clinical landscape, and clinical challenges of nanoparticle delivery systems. Here, we provide a 3 year update on the current clinical landscape of nanoparticle drug delivery systems and highlight newly approved nanomedicines, provide a status update on previous clinical trials, and highlight new technologies that have recently entered the clinic.

## 1 INTRODUCTION

The nanomedicine landscape continues to rapidly evolve driven by newly developed delivery strategies, new technologies, new treatment modalities, new drug approvals, and even clinical failures of current drugs. In 2016, we published a review article on the current clinical landscape of therapeutic nanoparticles, which highlighted over 25 Food and Drug Administration (FDA) or European Medicines Agency (EMA) approved nanomedicines and over 45 other nanoparticle technologies that were not FDA/EMA approved but were currently being evaluated in ongoing clinical trials.^1^ That article also featured discussions on different nanoparticle types, their applications, their advantages as compared to free drugs, and their potential. We also discussed many of the biological issues (i.e., biodistribution, biological barrier breaching, and treating heterogeneous diseases), technological issues (i.e., scale‐up limitations, parameter optimization, and predicting efficacy), and clinical challenges that have limited the translation of nanoparticles.^1^ In these past 3 years, since that article was published, two intravenously administered nanoparticles have been FDA and EMA approved, one intratumorally administered nanoparticle received European market approval (CE Mark), over 75 new trials have begun for the previously highlighted nonapproved nanoparticles, and over 15 new nanoparticle technologies have entered clinical trials. In this 3‐year update, we highlight these new clinical approvals, trials, and technologies to provide an updated snapshot on the current clinical landscape of nanoparticles in 2019.

## 2 NEW APPROVALS

Since our previous article, three nanomedicines have been approved: Patisiran/ONPATTRO, VYXEOS, and NBTXR3/Hensify. VYXEOS is a combination chemotherapy nanoparticle, developed and marketed by Jazz Pharmaceuticals that, encapsulates a synergistic molar ratio of cytarabine to daunorubicin of 5:1 and received FDA approval for the treatment of acute myeloid leukemia in August of 2017.^2^, ^3^ VYXEOS are 100 nm bilamellar liposomes where the lipid membrane consists of desaturated phosphatidylcholine:distearylphosphatidylglycerol:cholesterol (7:2:1M ratio).^4^ In the pivotal efficacy study (NCT01696084), VYXEOS provided a significant (*p* value = .005) improvement in overall survival of 9.6 months as compared to 5.9 months in the free drug control.^2^, ^5^ Importantly, this trial also showed that VYXEOS provided improved efficacy at a lower cumulative daunorubicin and cytarabine dose as compared to free drug counterparts.^6^ Since 2016, the number of clinical trials of VYXEOS has increased from 7 to 21 with the most recent trials investigating the use of VYXEOS in additional patient populations (e.g., children; NCT03826992) and leukemias (e.g., lymphoblastic leukemias; NCT03575325). Unlike other approved nanoparticles for cancer treatment, VYXEOS delivers two drugs in a synergistic ratio. Delivery of the synergistic combination of daunorubicin and cytarabine is enabled by the nanoparticle platform since the encapsulated ratio of drugs is able to both interact with target cells upon release. In the contrasting case of free drugs, each drug exhibits distinct pharmacokinetic profiles and are metabolized at different rates; as such, delivery of synergistic combinations of free drugs to target cells must also consider and counteract these biological processes. Product sales for VYXEOS were $100.8 million in 2018.^7^ As the first clinically approved nanoparticle to deliver a synergistic combination of free drugs, VYXEOS can pave the way for new combination nanoparticle formulations that leverage widely‐utilized combination chemotherapy regimens from the clinic.^8^, ^9^

Patisiran/ONPATTRO is an siRNA‐delivering lipid‐based nanoparticle developed and marketed by Alnylam, for the silencing of a specific gene responsible for expression of transthyretin, which can cause hereditary transthyretin amyloidosis.^10^ Patisiran/ONPATTRO lipid nanoparticles consist of (6Z,9Z,28Z,31Z)‐heptatriaconta‐6,9,28,31‐tetraen‐19‐yl‐4‐(dimethylamino) butanoate (DLin‐MC3‐DMA) plus cholesterol, 1,2‐distearoyl‐*sn*‐glycero‐3‐phosphocholine and α‐(3′‐{[1,2‐di(myristyloxy)propanoxy] carbonylamino}propyl)‐ω‐methoxy polyoxyethylene (PEG_2000_‐C‐DMG).^11^ Patisiran/ONPATTRO was approved by the FDA in August of 2018^12^ and was the first clinically approved example of an RNAi therapy‐delivering nanoparticle administered intravenously. Importantly, Patisiran/ONPATTRO is also the first FDA approved RNAi therapeutic in general,^12^ independent of the nanoparticle delivery vehicle. Approval of the first RNAi therapeutic was a major milestone in the biotech industry and considering that the delivery vehicle was a nanoparticle, approval of Patisiran/ONPATTRO was also a major milestone for nanomedicines. In the Phase III efficacy study (NCT01960348), 56% of patients receiving Patisiran/ONPATTRO exhibited improvements in modified Neuropathy Impairment Score+7 as compared to 4% receiving the placebo.^10^ Moreover, serum transthyretin decreased by over 70% in patients receiving Patisiran/ONPATTRO as compared to less than 20% in patients receiving the placebo.^10^ Global net revenues for Patisiran/ONPATTRO were $12.1 million in 2018 with over 200 patients in Europe and the United States receiving treatment.^13^ As the first clinically approved siRNA/RNAi therapeutic, Patisiran/ONPATTRO demonstrates how nanoparticles can be used to enable the delivery, and in this case approval, of highly challenging therapeutics to humans.

NBTXR3/Hensify is a 50 nm crystalline hafnium oxide nanoparticle with negatively charged phosphate coating, developed and marketed by Nanobiotix.^14^ NBTXR3/Hensify enhances external radiotherapy via a physical mode of action that relies on hafnium's natural radioenhancing properties.^14^, ^15^ Specifically, the interaction between ionizing radiation and hafnium facilitates a higher energy deposit as compared to ionizing radiation without hafnium interaction; this results in the generation of significantly more electrons and increases radiation‐mediated cell death from standard radiation oncology procedures.^14^, ^15^ NBTXR3/Hensify received CE Mark approval in April of 2019 for the treatment of locally advanced soft tissue sarcoma.^16^ Since our previous article, the number of clinical trials of NBTXR3/Hensify has increased from 1 to 8. While NBTXR3/Hensify is approved for intratumoral administration, clinical trials had investigated it for intra‐arterial administration (NCT01946867). The newest trials are only investigating NBTXR3/Hensify for intratumoral injections, but have expanded their indications to include treatment of prostate cancer (NCT02805894) and lung cancer with combined immunotherapy (NCT03589339). The reasoning for including immunotherapy with NBTXR3/Hensify treatment builds on preclinical data that demonstrated improved efficacy of immunotherapies following NBTXR3/Hensify treatment, stemming from an increased antitumor immune response.^17^, ^18^ Since the mechanism of action of NBTXR3/Hensify is unique and unlike other approved nanoparticles or therapeutics, NBTXR3/Hensify may represent the next‐generation of nanoparticle therapeutics; specifically, nanoparticle therapeutics that can provide therapeutic benefits in a complementary and possibly synergistic way to standard therapeutic modalities. Table 1, which previously listed FDA/EMA approved nanomedicines as of 2016, is now updated to include these recently approved nanoparticles.

**Table 1:** Updated clinically approved nanoparticle therapies and diagnostics, grouped by their broad indication

Name	Particle type/drug	Approved application/indication	Approval (year)	Investigated application/indication	Updates on number of studies on ClinicalTrials.gov identifier
New approvals since 2016					
VYXEOS CPX‐351 (Jazz Pharmaceuticals)	Liposomal formulation of cytarabine:daunorubicin (5:1M ratio)	Acute myeloid leukemia	FDA (2017) EMA (2018)	Various leukemias	2016: VYXEOS: 7 2019: VYXEOS: 21
ONPATTRO Patisiran ALN‐TTR02 (Alnylam Pharmaceuticals)	Lipid nanoparticle RNAi for the knockdown of disease‐causing TTR protein	Transthyretin (TTR)‐mediated amyloidosis	FDA (2018) EMA (2018)	Transthyretin (TTR)‐mediated amyloidosis	2016: 3 2019: 11
NBTXR3 Hensify (Nanobiotix)	Hafnium oxide nanoparticles stimulated with external radiation to enhance tumor cell death via electron production	Locally advanced squamous cell carcinoma	CE Mark (2019)	Locally advanced soft tissue sarcoma	2016: 1 (an additional trial was listed as completed at the time) 2019: 8
Cancer nanoparticle medicines					
Doxil Caelyx (Janssen)	Liposomal doxorubicin (PEGylated)	Ovarian cancer (secondary to platinum based therapies) HIV‐associated Kaposi's sarcoma (secondary to chemotherapy) Multiple myeloma (secondary)	FDA (1995) EMA (1996)	Various cancers including: solid malignancies, ovarian, breast, leukemia, lymphomas, prostate, metastatic, or liver	2016: Doxil: 166 CAELYX: 90 2019: Doxil: 182 CAELYX: 109
DaunoXome (Galen)	Liposomal daunorubicin (non‐PEGylated)	HIV‐associated Kaposi's sarcoma (primary)	FDA (1996)	Various leukemias	2016: DaunoXome: 32 2019: DaunoXome: 15
Myocet (Teva UK)	Liposomal doxorubicin (non‐PEGylated)	Treatment of metastatic breast cancer (primary)	EMA (2000)	Various cancers including: breast, lymphoma, or ovarian	2016: Myocet: 32 2019: Myocet: 35
Abraxane (Celgene)	Albumin‐particle bound paclitaxel	Advanced non‐small cell lung cancer (surgery or radiation is not an option) Metastatic breast cancer (secondary) Metastatic pancreatic cancer (primary)	FDA (2005) EMA (2008)	Various cancers including: solid malignancies, breast, lymphomas, bladder, lung, pancreatic, head and neck, prostate, melanoma, or liver	2016: Abraxane: 295 2019: Abraxane: 432
Marqibo (Spectrum)	Liposomal vincristine (non‐PEGylated)	Philadelphia chromosome‐negative acute lymphoblastic leukemia (tertiary)	FDA (2012)	Various cancers including: lymphoma, brain, leukemia, or melanoma	2016: Marqibo: 23 2019: Marqibo: 28
MEPACT (Millennium)	Liposomal mifamurtide (non‐PEGylated)	Treatment for osteosarcoma (primary following surgery)	EMA (2009)	Osteosarcomas	2016: MEPACT: 4 (3 active/recruiting) 2019: MEPACT: 9 (3 active/recruiting)
Onivyde MM‐398 (Merrimack)	Liposomal irinotecan (PEGylated)	Metastatic pancreatic cancer (secondary)	FDA (2015)	Various cancers including: solid malignancies, breast, pancreatic, sarcomas, or brain	2016: MM‐398/Onivyde: 7 (6 active/recruiting) 2019: MM‐398/Onivyde: 38 (26 active/recruiting)
Iron‐replacement nanoparticle therapies					
CosmoFer INFeD Ferrisat (Pharmacosmos)	Iron dextran colloid	Iron deficient anemia	FDA (1992) Some of Europe	Iron deficient anemia	2016: INFeD: 6 (1 recruiting) 2019: INFeD: 9
DexFerrum DexIron (American Regent)	Iron dextran colloid	Iron deficient anemia	FDA (1996)	Iron deficient anemia	2016: DexFerrum: 6 2019: DexFerrum: 9
Ferrlecit (Sanofi)	Iron gluconate colloid	Iron replacement for anemia treatment in patients with chronic kidney disease	FDA (1999)	Iron deficient anemia	2016: Ferrlecit: 13 (2 recruiting) 2019: Ferrlecit: 20 (0 recruiting)
Venofer (American Regent)	Iron sucrose colloid	Iron replacement for anemia treatment in patients with chronic kidney disease	FDA (2000)	Iron deficient anemia Following autologous stem cell transplantation	2016: Venofer: 44 2019: Venofer: 60
Feraheme (AMAG) Rienso (Takeda) Ferumoxytol	Iron polyglucose sorbitol carboxymethylether colloid	Iron deficiency in patients with chronic kidney disease	FDA (2009)	Iron deficient anemia Imaging: brain metastases, lymph node metastases, neuroinflammation in epilepsy, head and neck cancer, myocardial infarction, or multiple sclerosis	2016: Ferumoxytol: 57 (6 recruiting/active for anemia treatment; 22 recruiting/active for imaging applications) 2019: Ferumoxytol: 84 (6 recruiting/active for anemia treatment; 22 recruiting/active for imaging applications)
Injectafer Ferinject (Vifor)	Iron carboxymaltose colloid	Iron deficient anemia	FDA (2013)	Iron deficient anemia	2016: Ferinject: 50 Injectafer: 8 2019: Ferinject: 79 Injectafer: 24
Monofer (Pharmacosmos)	10% iron isomaltoside 1,000 colloid	Treating iron deficiency and anemia when oral methods do not work or when iron delivery is required immediately	Some of Europe	Iron deficient anemia	2016: Monofer: 22 (3 active/recruiting) 2019: Monofer: 22 (11 active/recruiting)
Diafer (Pharmacosmos)	5% iron isomaltoside 1,000 colloid	Iron deficient anemia	Some of Europe	Iron deficient anemia	2016: Diafer: 1 recruiting 2019: Diafer: 1 completed
Nano/microparticle imaging agents					
Definity (Lantheus Medical Imaging)	Perflutren lipid microspheres	Ultrasound contrast agent	FDA (2001)	Ultrasound enhancement for: liver or breast or intraocular or pancreatic tumors, pulmonary diseases, heart function, transcranial injuries, strokes, or liver cirrhosis	2016: Definity: 58 2019: Definity: 87
Feridex I.V. (AMAG) Endorem	Iron dextran colloid	Imaging of liver lesions	FDA (1996) Discontinued (2008)	N/A: No current studies	2016: Endorem: 4 Feridex: 2 No current active or recruiting studies 2019: Endorem: 4 Feridex: 2 No current active or recruiting studies
Optison (GE Healthcare)	Human serum albumin stabilized perflutren microspheres	Ultrasound contrast agent	FDA (1997) EMA (1998)	Ultrasound enhancement for: lymph node, renal cell carcinoma, myocardial infarction, pulmonary transit times, or heart transplant rejections	2016: Optison: 11 currently active or recruiting studies 2019: Optison: 30 (6 active)
SonoVue (Bracco Imaging)	Phospholipid stabilized microbubble	Ultrasound contrast agent	EMA (2001)	Ultrasound enhancement for: liver neoplasms, prostate or breast or pancreatic cancer, or coronary/pulmonary disease	2016: SonoVue: 43 2019: SonoVue: 72
Resovist (Bayer Schering Pharma) Cliavist	Iron carboxydextran colloid	Imaging of liver lesions	Some of Europe Discontinued (2009)	N/A No current studies	2016: 2 studies mention Resovist: No current active or recruiting studies 2019: 2 studies mention Resovist: No current active or recruiting studies
Ferumoxtran‐10 Combidex Sinerem (AMAG)	Iron dextran colloid	Imaging lymph node metastases	Only available in Holland	Imaging lymph node metastases	2016: Ferumoxtran‐10:11 (1 active) 2019: Ferumoxtran‐10:24 (1 active; 6 recruiting)
Nanoparticle vaccines					
Epaxal (Crucell)	Liposome with hepatitis A virus	Hepatitis A vaccine	Some of Europe (discontinued)	Safety and immunogenicity of hepatitis A vaccine	2016: Epaxal: 6 (1 recruiting) 2019: Epaxal: 6 (0 recruiting)
Inflexal V (Crucell)	Liposome with trivalent‐influenza	Influenza vaccine	Some of Europe (discontinued)	Safety and immunogenicity of influenza vaccine	2016: Inflexal V: 14 (all completed) 2019: Inflexal V: 14 (all completed)
Particle anesthetics					
Diprivan	Liposomal propofol	Induction and maintenance of sedation or anesthesia	FDA (1989)	General anesthesia in specific situations: morbidly obese patients, open heart surgery, or spinal surgery	2016: Diprivan: 110 2016: Diprivan: 162
Nanoparticles for fungal treatments					
AmBisome (Gilead Sciences)	Liposomal amphotericin B	Cryptococcal meningitis in HIV‐infected patients Aspergillus, Candida and/or Cryptococcus species infections (secondary) Visceral leishmaniasis parasite in immunocompromised patients	FDA (1997) Most of Europe	Preventing or treating invasive fungal infections	2016: AmBisome: 50 2019: AmBisome: 57
Nanoparticles for macular degeneration					
Visudyne (Bausch and Lomb)	Liposomal verteporfin	Treatment of subfoveal choroidal neovascularization from age‐related macular degeneration, pathologic, or ocular histoplasmosis	FDA (2000) EMA (2000)	Macular degeneration	2016: Visudyne: 52 2016: Visudyne: 60

*Note*: Newly approved nanoparticles are separately listed in the first rows. Modified with permission from Reference 1.

Abbreviations: EMA, European Medicines Agency; FDA, Food and Drug Administration.

## 3 UPDATE ON PREVIOUS TRIALS

In our previous article, over 45 different nonapproved nanoparticles (liposomes, polymeric, micelles, albumin‐bound nanoparticles, and inorganic nanoparticles) were listed as active in a total of over 80 different clinical trials (mostly for the treatment of various cancers but also radiation exposure, arthritis, pneumonia, amyloidosis, hepatitis, and fibrosis). Of these 80 trials, 28 have since been completed with 12 being terminated early. Of the 45 different nanoparticles, seven possessed targeting functionality, and six offered stimuli‐responsive functions (e.g., thermal ablation in response to near‐infrared light, thermosensitive liposomes). Three of these nanoparticles, as mentioned above, have received FDA, EMA, or CE Mark approval. Here, we have updated our previous table to reflect the current status of each of these technologies to include new clinical trials and updates on previous trials. Seventy‐five new trials exist for the previously highlighted nanoparticles. Of these 75 new trials, 14 are for VYXEOS, 8 are for Patisiran/ONPATTRO, and 6 are for NBTXR3/Hensify. Of particular note, CRLX101, a cyclodextrin‐based nanoparticle‐camptothecin conjugate, began nine new trials and ABI‐009, albumin bound rapamycin, began 12 new trials. Table 2 summarizes these findings and additionally provides technical and clinical updates, when publicly available, for these clinically investigated nanoparticles.

**Table 2:** Updates on previously reported intravenous nanoparticle clinical trials that have not been clinically approved and are currently undergoing clinical trials (not yet recruiting, recruiting, or active)

Name (company)	Particle type/drug	Investigated application/indication	ClinicalTrials.gov identifiers (phase)	Updates since 2016
Liposomes (cancer)				
PROMITIL (Lipomedix Pharmaceuticals)	PEGylated liposomal mitomycin‐C	Solid tumors	2016: NCT01705002 (Ph I): Completed 2019 additions: NCT03823989 (Ph Ib): Recruiting	1 new trial 1 trial completed
ThermoDox® (Celsion)	Lyso‐thermosensitive liposomal doxorubicin	Temperature‐triggered doxorubicin release: Breast cancer recurrence at chest wall (microwave hypothermia) Hepatocellular carcinoma (radiofrequency ablation) Liver tumors (mild hypothermia) Refractory solid tumors (magnetic resonance high intensity focused ultrasound)	2016: NCT02536183 (Ph I): Recruiting NCT00826085 (Ph I/II): Completed NCT02112656 (Ph III): Completed NCT02181075 (Ph I): Completed 2019 additions: NCT03749850 (Ph I): Not yet recruiting	1 new trial 3 trials completed NCT02181075 (Ph I): Published results highlight how ThermoDox in combination with externally induced mild hyperthermia increase intratumoral concentration of dox by 3.7 times as compared to ThermoDox without hyperthermia induction.^26^
VYXEOS CPX‐351 (Celator Pharmaceuticals)	Liposomal formulation of cytarabine:daunorubicin (5:1M ratio)	Leukemias	2016: NCT01804101 (not provided) NCT02286726 (Ph II) NCT02019069 (Ph II) NCT01943682 (Ph I) NCT02269579 (Ph II) NCT02533115 (Ph IV) NCT01696084 (Ph III) 2019 additions: 21 Total studies	Received FDA approval in 2017 and EMA approval in 2018 13 new trials
Oncoprex (Genprex)	FUS1 (TUSC2) encapsulated liposome	Lung cancer	2016: NCT01455389 (Ph I/II): Active, not recruiting	0 new trials
Halaven E7389‐LF (Eisai)	Liposomal eribulin mesylate	Solid tumors	2016: NCT01945710 (Ph I): Completed 2019 additions: NCT03207672 (Ph I): Recruiting	1 new trial 1 trial completed
^188^Re‐BMEDA‐liposome	^188^Re‐N,N‐bis (2‐mercaptoethyl)‐N′,N′‐diethylethylenediamine pegylated liposome	Advanced solid tumors	2016: NCT02271516 (Ph I): Unknown	0 new trials
Mitoxantrone hydrochloride liposome (CSPC ZhongQi Pharmaceutical Technology)	Mitoxantrone liposome	Lymphoma and breast cancer	2016: NCT02131688 (Ph I): Unknown NCT02596373 (Ph II): Recruiting NCT02597387 (Ph II): Recruiting NCT02595242 (Ph I): Withdrawn NCT02597153 (Ph II): Terminated (only one subject enrolled in 1.5 years) 2019 additions: NCT03776279 (Ph I): Recruiting	1 new trial 1 trial withdrawn 1 trial terminated
JVRS‐100	Cationic liposome incorporating plasmid DNA complex for immune system stimulation	Leukemia	2016: NCT00860522 (Ph I): Completed	0 new trials 1 trial completed
Lipocurc (SignPath Pharma)	Liposomal curcumin	Solid tumors	2016: NCT02138955 (Ph I/II): Unknown	0 new trials 1 trial changed to unknown status
LiPlaCis (LiPlasome Pharma)	Liposomal formulated cisplatin with specific degradation‐controlled drug release via phospholipase A2 (PLA2)	Advanced or refractory tumors	2016: NCT01861496 (Ph I): Recruiting	0 new trials
MM‐302 (Merrimack Pharmaceuticals)	HER2‐targeted liposomal doxorubicin (PEGylated)	Breast cancer	2016: NCT01304797 (Ph I): Unknown NCT02213744 (Ph II/III): Terminated (felt not to show benefit over control per DMC and confirmed via futility analysis) 2019 additions: NCT02735798 (Ph I): Withdrawn (the study was not started due to the sponsor choosing to not fund the trial)	1 new trial that was withdrawn 1 trial terminated 1 trial changed to unknown status Merrimack halted the phase II study of MM‐302 (NCT02213744) due to it being unlikely that MM‐302 would demonstrate benefits over the control comparison.^27^ Merrimack published results for NCT01304797 where data suggested that a tracer nanoparticle could be used to select for patients that exhibit enhanced EPR effect as a means to screen for patients who would likely respond favorably to nanomedicines.^28^
LIPUSU® (Nanjing Luye Sike Pharmaceutical Co., Ltd.)	Paclitaxel liposome	Advanced solid tumors, or gastric, breast cancer	2016: NCT01994031 (Ph IV): Unknown NCT02142790 (Ph IV): Unknown NCT02163291 (Ph II): Unknown NCT02142010 (not provided): Unknown 2019 additions: NCT02996214 (Ph IV): Not yet recruiting	1 new trial
Liposomes (gene therapy: Cancer)				
TKM‐080301 (Arbutus Biopharma)	Lipid particle targeting polo‐like kinase 1 (PLK1) for delivery of siRNA	Hepatocellular carcinoma	2016: NCT02191878 (Ph I/II): Completed	0 new trials 1 trial completed
siRNA‐EphA2‐DOPC	siRNA liposome for EphA2 knockdown	Solid tumors	2016: NCT01591356 (Ph I): Recruiting	0 new trials
PNT2258 (ProNAi Therapeutics)	Proprietary single‐stranded DNAi (PNT100) encapsulated in lipid nanoparticles	Lymphomas	2016: NCT02378038 (Ph II): Terminated NCT02226965 (Ph II): Unknown NCT01733238 (Ph II): Completed	0 new trials 1 trial completed 1 trial terminated 1 trial changed to unknown status
BP1001 (Bio‐Path Holdings)	Growth factor receptor bound protein‐2 (Grb‐2) antisense oligonucleotide encapsulated in neutral liposomes	Leukemias	2016: NCT01159028 (Ph I): Active, not recruiting 2019 additions: NCT02923986 (Ph I): Recruiting NCT02781883 (Ph II): Recruiting	2 new trials
DCR‐MYC (Dicerna Pharmaceuticals)	DsiRNA lipid nanoparticle for NYC oncogene silencing	Solid tumors, multiple myeloma, lymphoma, or hepatocellular carcinoma	2016: NCT02110563 (Ph I): Terminated (sponsor decision) NCT02314052 (Ph I/II) terminated (sponsor decision)	0 new trials 2 trials terminated DCR‐MYC development discontinued.^29^
Atu027 (Silence Therapeutics GmbH)	AtuRNAi liposomal formulation for PKN3 knockdown in vascular endothelium	Pancreatic cancer	2016: NCT01808638 (Ph I/II): Completed	0 new trials 1 trial completed
SGT‐53 (SynerGene Therapeutics)	Cationic liposome with anti‐transferrin receptor antibody, encapsulating wildtype p53 sequence	Glioblastoma, solid tumors, or pancreatic cancer	2016: NCT02354547 (Ph I): Recruiting NCT02354547 (Ph I): Recruiting NCT02340156 (Ph II): Recruiting NCT00470613 (Ph I): Completed 2019 additions: NCT03554707 (Ph I): Not yet recruiting	1 new trial 1 trial completed
SGT‐94 (SynerGene Therapeutics)	RB94 plasmid DNA in a liposome with anti‐transferrin receptor antibody	Solid tumors	2016: NCT01517464 (Ph I): Completed	0 new trials 1 trial completed
MRX34 (Mirna Therapeutics)	Double‐stranded RNA mimic of miR‐34 encapsulated in liposomes	Liver cancer	2016: NCT01829971 (Ph I): Terminated (five immune related serious adverse events) 2019 additions: NCT02862145 (Ph I): Withdrawn (5 immune related serious adverse events in phase 1 study)	1 new trial that was withdrawn 1 trial terminated
TargomiRs (EnGeneIC)	Anti‐EGFR bispecific antibody minicells (bacteria derived nanoparticles) with a miR‐16 based microRNA payload	Mesothelioma and non‐small cell lung cancer	2016: NCT02369198 (Ph I): Completed	0 new trials 1 trial completed NCT02369198 (Ph I): Published study demonstrates that TargomiRs were well‐tolerated by refractory malignant pleural mesothelioma patients.^30^
Liposomes (gene therapy: Other)				
ND‐L02‐s0201 (Nitto Denko)	siRNA lipid nanoparticle conjugated to vitamin A	Hepatic fibrosis and pulmonary fibrosis	2016: NCT02227459 (Ph I): Completed 2019 additions: NCT01858935 (Ph I): Completed NCT03241264 (Ph I): Completed NCT03538301 (Ph II): Recruiting	3 new trials (2 completed) 1 trial completed
ARB‐001467 TKM‐HBV (Arbutus Biopharma)	Lipid particle containing three RNAi therapeutics that target three sites on the HBV genome	Hepatitis B	2016: NCT02631096 (Ph II): Completed	0 new trials 1 trial completed
ONPATTRO Patisiran ALN‐TTR02 (Alnylam Pharmaceuticals)	Lipid nanoparticle RNAi for the knockdown of disease‐causing TTR protein	Transthyretin (TTR)‐mediated amyloidosis	2016: NCT02510261 (Ph III) NCT01961921 (Ph II) NCT01960348 (Ph III) 2019 additions: 11 total studies	Received FDA and EMA approval in 2018
Liposomes (other)				
CAL02 (Combioxin SA)	Sphingomyelin and cholesterol liposomes for toxin neutralization	Pneumonia	2016: NCT02583373 (Ph I): Completed	0 new trials 1 trial completed
Nanocort (Enceladus in collaboration with sun pharma global)	Liposomal prednisolone (PEGylated)	Rheumatoid arthritis and hemodialysis fistula maturation	2016: NCT02495662 (Ph II): Terminated (slow inclusion) NCT02534896 (Ph III): Terminated	0 new trials 2 trials terminated
RGI‐2001 (Regimmune)	Liposomal formulaton of α‐GalCer	Mitigating graft versus host disease following stem cell transplant	2016: NCT01379209 (Ph I/II): Unknown 2019 additions: NCT04014790 (Ph II): Not yet recruiting	1 new trial
Sonazoid	F‐butane encapsulated in a lipid shell	Contrast enhanced ultrasound for imaging hepatocellular carcinoma, skeletal muscle perfusion, or for estimating portal hypertension	2016: NCT00822991 (not provided): Recruiting NCT02398266 (Ph II): Unknown NCT02188901 (not provided): Completed NCT02489045 (Ph IV): Recruiting	0 new trials 1 trial changed to unknown status 1 trial completed
Polymeric and micelles (cancer)				
AZD2811 (AstraZeneca with BIND Therapeutics)	Aurora B kinase inhibitor in BIND therapeutics polymer particle accurin platform	Advanced solid tumors	2016: NCT02579226 (Ph I): Recruiting 2019 additions: NCT03366675 (Ph II): Terminated (early detection of the purpose of the study) NCT03217838 (Ph I): New, recruiting	2 new trials (1 terminated)
BIND‐014 (BIND Therapeutics)	PSMA targeted (via ACUPA) docetaxel PEG‐PLGA or PLA–PEG particle	Prostate, metastatic, non‐small cell lung, cervical, head and neck, or KRAS positive lung cancers	2016: NCT02479178 (Ph II): Terminated NCT02283320 (Ph II): Completed NCT01812746 (Ph II): Completed NCT01792479 (Ph II): Completed NCT01300533 (Ph I): Completed	0 new trials 4 trials completed Pfizer purchased BIND Therapeutics' bankruptcy assets July 2016.^31^
Cynviloq IG‐001 (Sorrento)	Paclitaxel polymeric micelle nanoparticle	Breast cancer	2016: NCT02064829 (not provided): Completed	0 new trials 1 trial completed
Genexol‐PM (Samyang Biopharmaceuticals)	Paclitaxel polymeric micelle nanoparticle	Head and neck or breast cancer	2016: NCT01689194 (Ph II): Unknown NCT02263495 (Ph II): Completed NCT00912639 (Ph IV): Unknown 2019 additions: NCT02739633 (Ph II): Recruiting NCT03008512 (Ph II): Recruiting	2 new trials 1 trial completed 1 trial changed to unknown status
NC‐6004 Nanoplatin (Nanocarrier)	Polyamino acid, PEG, and cisplatin derivative micellar nanoparticle	Advanced solid tumors, lung, biliary, bladder, or pancreatic cancers	2016: NCT02240238 (Ph I/II): Active, not recruiting NCT02043288 (Ph III): Unknown 2019 additions: NCT03771820 (Ph II): Not yet recruiting NCT03109158 (Ph I): Completed NCT02817113 (Ph I): Unknown	3 new trials 1 trial changed to unknown status
NC‐4016 DACH‐Platin micelle (Nanocarrier)	Polyamino acid, PEG, and oxaliplatin micellar nanoparticle	Advanced solid tumors or lymphomas	2016: NCT01999491 (Ph I): Completed	0 new trials
NK105 (Nippon Kayaku)	Paclitaxel micelle	Breast cancer	2016: NCT01644890 (Ph III): Completed	0 new trials 1 trial completed
Docetaxel‐PM DOPNP201 (Samyang Biopharmaceuticals)	Docetaxel micelle	Head and neck cancer and advanced solid tumors	2016: NCT02639858 (Ph II): Recruiting NCT02274610 (Ph I): Completed 2019 additions: NCT03585673 (Ph II): Recruiting	1 new trial 1 trial completed
CriPec (Cristal Therapeutics)	Docetaxel micelles	Solid tumors, ovarian cancer	2016: NCT02442531 (Ph I): Completed 2019 additions: NCT03712423 (Ph I): Recruiting NCT03742713 (Ph II): Recruiting	2 new trials 1 trial completed
CRLX101 (Cerulean)	Cyclodextrin‐based nanoparticle‐camptothecin conjugate	Ovarian, renal cell, small cell lung, or rectal cancers	2016: NCT02187302 (Ph II): Completed NCT02010567 (Ph I/II): Active, not recruiting NCT02389985 (Ph I): Terminated (company decision) NCT01803269 (Ph II): Terminated (due to lack of activity and slow accrual) NCT01652079 (Ph II): Completed 2019 additions: NCT02769962 (Ph I): Recruiting NCT03531827 (Ph II): Recruiting NCT02648711(Ph I): Terminated (company decision) NCT01380769 (Ph II): Completed NCT01612546 (Ph II): Completed NCT00333502 (Ph II): Completed NCT01625936 (Ph I): Completed NCT00753740 (Ph II): Withdrawn (poor trial recruitment) NCT00163319 (Ph III): Completed	9 new trials (1 terminated, 1 withdrawn, 5 completed) 2 previous trials completed 2 previous trials terminated NCT02010567 (Ph I/II): Addition of CRLX101 to standard chemoradiotherapy was in locally advanced rectal cancer patients demonstrated well‐tolerability.^32^
CRLX301 (Cerulean)	Cyclodextrin based nanoparticle‐docetaxel conjugate	Dose escalation study in advanced solid tumors	2016: NCT02380677 (Ph I/II): Terminated (company decision)	0 new trials 1 trial terminated
Polymeric and micelles (other)				
RadProtect (Original BioMedicals)	PEG, iron, and amifostine micelle Transferrin‐mediated chelation for amifostine release	Dose escalation and safety for acute radiation syndrome	2016: NCT02587442 (Ph I): Unknown	0 new trials
Albumin‐bound (cancer)				
ABI‐009 (Aadi with Celgene)	Albumin bound rapamycin	Bladder cancer, PEComa, or pulmonary arterial hypertension	2016: NCT02009332 (Ph I/II): Recruiting NCT02587325 (Ph I): Recruiting NCT02494570 (Ph II): Active not recruiting 2019 additions: NCT03747328 (Ph II): Not yet recruiting NCT03657420 (Ph I): Not yet recruiting NCT03670030 (Ph II): Recruiting NCT03646240 (Ph I): Recruiting NCT03190174 (Ph I): Recruiting NCT00635284 (Ph I): Completed NCT03817515: Expanded access status: Available NCT03439462 (Ph II): Recruiting NCT03463265 (Ph II): Recruiting NCT03660930 (Ph I): Recruiting NCT02975882 (Ph I): Recruiting NCT02646319 (Ph I): Completed	12 new trials (2 completed)
ABI‐011 (NantBioScience)	Albumin bound thiocolchicine analog (IDN 5405)	Solid tumors or lymphomas	2016: NCT02582827 (Ph I): Recruiting	0 new trials
Inorganic (cancer)				
AuroLase (Nanospectra Biosciences)	PEG‐coated silica‐gold nanoshells for near infrared light facilitated thermal ablation	Thermal ablation of solid primary and/or metastatic lung tumors	2016: NCT01679470 (not provided): Terminated 2019 additions: NCT02680535 (not provided): Recruiting NCT00848042 (not provided): Completed	2 new trials (1 completed) 1 trial terminated
NBTXR3 PEP503 (Nanobiotix)	Hafnium oxide nanoparticles stimulated with external radiation to enhance tumor cell death via electron production	Locally advanced squamous cell carcinoma	2016: NCT01946867 (Ph I): Unknown 2019 additions: NCT02721056 (Ph II): Unknown NCT02805894 (Ph II): Recruiting NCT03589339 (Ph II): Not yet recruiting NCT02379845 (Ph II): Active not recruiting NCT02901483 (Ph I): Recruiting NCT02465593 (Ph I): Recruiting	Received CE mark approval in 2019 6 new trials 1 trial changed to unknown status
Cornell Dots	Silica nanoparticles with a NIR fluorophore, PEG coating, and a ^124^I radiolabeled cRGDY targeting peptide	Imaging of melanoma and malignant brain tumors	2016: NCT01266096 (not provided): Active, not recruiting 2019 additions: NCT03465618 (Ph I): Recruiting NCT02106598 (Ph II): Recruiting	2 new trials
Magnablate	Iron nanoparticles	Thermal ablation for prostate cancer	2016: NCT02033447 (Ph 0): Completed	0 new trials 1 trial completed

*Note*: These trials are grouped by particle type and inidication. Modified with permission from Reference 1.

Abbreviations: EMA, European Medicines Agency; FDA, Food and Drug Administration.

## 4 NEW NANOPARTICLE TRIALS

Since 2016, our search revealed 18 new nanoparticles to have entered clinical trials. Of these 18 nanoparticles, 12 are liposomes and 17 are indicated for cancer (15 being for treatment and 2 for imaging). The lone non‐cancer indication is mRNA‐1944, which are two mRNAs encoding heavy and light chains of anti‐Chikungunya antibody formulated in lipid nanoparticles, toward the prevention of Chikungunya virus infection. Table 3 summarizes these findings. It should be noted that other clinical trials investigating nanoparticles for the delivery of mRNA exist but since they are predominately delivered through intradermal or other routes of administration they will not be covered here. We point the reader to a recent review on mRNA delivery strategies where current clinical trials and delivery vehicles are a primary focus.^19^

**Table 3:** Intravenous nanoparticle therapies and diagnostics which are not clinically approved and are currently undergoing clinical trials (not yet recruiting, recruiting, enrolling by invitation, or active)

Name (company)	Particle type/drug	Investigated application/indication	Current ClinicalTrials.gov identifiers (phase)
Liposomes (cancer)			
MM‐310 (Merrimack Pharmaceuticals)	Nanoliposomal encapsulated docetaxel and functionalized with antibodies targeted to the EphA2 receptor	Solid tumors	NCT03076372 (Ph I): Recruiting
EGFR(V)‐EDV‐Dox (EnGeneIC)	Bacterially derived minicell encapsulating doxorubicin	Recurrent glioblastoma	NCT02766699 (Ph I): Recruiting
Alprostadil liposome (CSPC ZhongQi Pharmaceutical Technology)	Alprostadil liposome	Safety and tolerability	NCT03669562 (Ph I): Recruiting
Liposomal Annamycin (Moleculin Biotech)	Liposomal Annamycin	Acute myeloid leukemia	NCT03388749 (Ph II): Recruiting NCT03315039 (Ph II): Recruiting
FF‐10831 (Fujifilm Pharmaceuticals)	Liposomal Gemcitabine	Advanced solid tumors	NCT03440450 (Ph I): Recruiting
Anti‐EGFR‐IL‐dox (Swiss Group for Clinical Cancer Research; University Hospital, Basel, Switzerland)	Doxorubicin‐loaded anti‐EGFR immunoliposomes	Advanced triple negative EGFR positive breast cancer High grade gliomas	NCT02833766 (Ph II): Recruiting NCT03603379 (Ph I): Recruiting
TLD‐1/Talidox (InnoMedica)	A new formulation of liposomal doxorubicin	Advanced solid tumors	NCT03387917 (Ph I): Recruiting
NC‐6300 (NanoCarrier)	Micelle encapsulated epirubicin	Advanced solid tumors or soft tissue sarcoma	NCT03168061 (Ph II): Recruiting
Liposomes (gene therapy: Cancer)			
MRT5201 (Translate Bio)	mRNA encapsulated in PEGylated liposomes	Ornithine transcarbamylase deficiency	NCT03767270 (Ph I): Not yet recruiting
Lipo‐MERIT (Biontech RNA Pharmaceuticals)	Four naked ribonucleic acid (RNA)‐drug products formulated with liposomes	Cancer vaccine for advanced melanoma	NCT02410733 (Ph I): Recruiting
Liposomes (immunotherapy: Cancer)			
IVAC_W_bre1_uID	Patient‐specific liposome (specificity for antigen‐expression on a patient's tumor) complexed RNA	Triple negative breast cancer	NCT02316457 (Ph I): Recruiting
Liposomes (gene therapy: Vaccine)			
mRNA‐1944 (Moderna)	Two mRNAs that encode heavy and light chains of anti‐Chikungunya antibody formulated in Moderna's proprietary lipid nanoparticle technology	Safety, tolerability, pharmacokinetics and pharmacodynamics towards the prevention of Chikungunya virus infection	NCT03829384 (Ph I): Recruiting
Micelles (cancer)			
MTL‐CEBPA (Mina alpha)	Double stranded RNA formulated into SMARTICLES amphoteric liposomes	Advanced liver cancer	NCT02716012 (Ph I): Recruiting
Imx‐110 (Immix Biopharma Australia)	Micelle encapsulating a Stat3/NF‐kB/poly‐tyrosine kinase inhibitor and low‐dose doxorubicin	Advanced solid tumors	NCT03382340 (Ph I): Recruiting
IT‐141 (Intezyne Technologies)	Micelle formulation of SN‐38	Advanced cancer	NCT03096340 (Ph I): Recruiting
Inorganic nanoparticles (cancer)			
NU‐0129 (Northwestern)	Spherical nucleic acid platform consisting of nucleic acids arranged on the surface of a spherical gold nanoparticle	Glioblastoma	NCT03020017 (Ph I): Active, not recruiting
Nanoparticles for imaging applications			
AGuIX (National Cancer Institute, France)	Polysiloxane Gd‐Chelates‐based nanoparticles	Advanced cervical cancer	NCT03308604 (Ph I): Recruiting
ONM‐100 (OncoNano Medicine)	Micelle covalently conjugated to indocyanine green	Intraoperative detection of cancer	NCT03735680 (Ph II): Not yet recruiting

*Note*: These trials and nanoparticles have appeared on the ClinicalTrial.gov database since 2016. Trials are grouped by particle type and indication.

## 5 CONCLUSIONS

Nanoparticle drug delivery systems offer many advantages over their free drug counterparts, can fundamentally change how therapeutics are delivered, and also enable the development of novel treatment modalities. This is demonstrated by the recent approvals of Patisiran/ONPATTRO (the first FDA approved RNAi therapeutic), VYXEOS (a nanoparticle capable of delivering synergistic ratios of two drugs), and NBTXR3/Hensify (a radio‐enhancing nanoparticle that synergizes with standard of care radiation oncology treatments). On the other hand, nanoparticles also face unique challenges related to their biological, technological, and clinical limitations that must be addressed to achieve consistent clinical impact. These advantages and challenges were discussed in‐depth in the 2016 review^1^ and in many other reviews.^20^, ^21^, ^22^, ^23^, ^24^, ^25^ With the increasing numbers of nanoparticle clinical trials, including nanoparticle technologies that were in trials at the time of our previous article (Table 2) and those that have entered the clinic since then (Table 3), the interest and pursuit of successful nanoparticle technologies continues. Taken together with these recent approvals, the field of nanoparticle drug delivery continues to make breakthroughs that improve human health.
